# The Role of Lipid Transfer Proteins as Food and Pollen Allergens Outside the Mediterranean Area

**DOI:** 10.1007/s11882-020-00982-w

**Published:** 2021-02-03

**Authors:** Stephan Scheurer, Ronald van Ree, Stefan Vieths

**Affiliations:** 1 Molecular Allergology, Paul-Ehrlich-Institut, Paul-Ehrlich Str. 51-59, 63225 Langen, Germany; 2 Departments of Experimental Immunology and of Otorhinolaryngology, Amsterdam UMC, University of Amsterdam, Amsterdam, Netherlands

**Keywords:** Non-specific lipid transfer protein, LTP, LTP syndrome, Pru p 3, Mugwort

## Abstract

**Purpose of Review:**

To provide an overview of the prevalence and clinical manifestation of non-specific lipid transfer proteins (LTP)-mediated allergies outside the Mediterranean area and to address potential reasons for the different geographical significance of LTP-driven allergies.

**Recent Findings:**

LTPs are major allergens in the Mediterranean area, which frequently can elicit severe reactions. Pru p 3 the LTP from peach is reported as genuine allergen and is considered a prototypic marker for LTP-mediated allergies. However, both food and pollen LTP allergies exist outside the Mediterranean area, but with lower clinical significance, different immunogenicity, and less clarified role.

**Summary:**

Evidence has been reported that in areas with high exposure to pollen, in particular to mugwort, pollen-derived LTPs can act as a primary sensitizer to trigger secondary food allergies. Co-sensitization to unrelated allergens might be causative for less severe reactions in response to LTPs. However, the reason for the geographical different sensitization patterns to LTPs remains unclear.

## Introduction

Non-specific lipid transfer proteins (LTPs) are ubiquitous in terrestrial plants and described as pan allergens in plant-derived food (*Rosaceae* fruits, vegetables), as well as in tree (plane and olive) and weed (pellitory, ragweed, and mugwort) pollen. Potentially, any antigen can elicit an allergic response, but only a limited number of allergens and restricted number of protein families cause the majority of allergic reactions. Although allergens seem to display an intrinsic T_H_2-inducing immunogenicity, geographical differences for the prevalence of specific allergies are well described, e.g., for peanut allergy (in the USA in comparison to Europe), the birch food syndrome (in Central/Northern Europe, but not in the Mediterranean area), or the LTP syndrome (in Southern Europe, but, e.g., not in Scandinavia). Differences in the allergic sensitization can be associated with a different level of exposure to the respective allergens, e.g., to inhalant allergens due to pollen endemic areas and, depending on the climate condition, to food allergens due to different dietary habits, food processing techniques applied, and the age of children when foods are introduced to the diet. It is tempting to speculate that other factors such as the genetic background of individuals, the life style including rural or urban housing, the co-incidence with microbial and parasite infections, potential concomitant respiratory and immune modifying diseases, or the sensitization profile will contribute to a different geographical significance of certain allergies.

Allergic reactions to non-specific lipid transfer proteins (LTPs) from plant pollen and food predominantly occur in the Mediterranean area and are rare in the Northern and Central Europe (reviewed in [1]). In Southern Europe, sensitization to LTPs is dominated by peach Pru p 3: IgE responses to LTP are (almost) never seen without it, and IgE titers are usually the highest among those against LTPs. These observations are at the basis of the established consensus that peach is the primary sensitizer for Mediterranean LTP-driven allergy. Because of its high clinical significance, Pru p 3 is considered the prototypic marker for the LTP syndrome in this geographical area. To a lesser extent, LTP-mediated allergies also occur outside the Mediterranean, but the reasons for this geographic difference in the sensitization prevalence are still unknown.

The present review addresses differences between the established Mediterranean “cradle” of the LTP syndrome and other geographical locations, with particular attention on the clinical impact of co- and cross-sensitization patterns to pollen.

## Molecular Features and Properties of LTPs

Non-specific LTPs are ubiquitous plant proteins which are engaged in lipid membrane biosynthesis and which act as pathogenesis-related proteins (PR-14) [2, 3]. Along with the structurally closely related 2S-albumins and α-amylase/protease inhibitors, LTPs belong to the prolamin protein superfamily. Although LTPs are found in different phylogenetic plant families, and some LTPs from different plant species display less than 30% amino acid sequence identity (aa-id), the tertiary structure of LTPs defining conformational IgE epitopes is highly conserved. Due to their conserved expression in phylogenetically distantly related plant species and the strong cross-reactivity of IgE between LTPs, they are termed pan allergens.

The 3D structure is stabilized by four intramolecular di-sulfide bonds which confer high stability, resulting in resistance to proteolytic digestion and thermal processing in case of food LTPs. These features allow allergens belonging to this family to reach effector cells in a non-fragmented and IgE-reactive form, capable to evoke severe systemic allergic symptoms. In general, the frequency of systemic reactions in response to LTP likely is higher in comparison to Bet v 1-like proteins and profilin, but seems to depend on the patients in terms of the age and geographical area. Two allergenic LTP subfamilies have been described, LTP1 (9 kDa) and LTP2 (6 kDa), with most allergens belonging to the LTP1 family. In the Mediterranean area, Pru p 3, the LTP from peach, is considered in the majority of cases the genuine sensitizer for LTP-mediated allergies and causative for the LTP syndrome.

## Prevalence of Food and Pollen LTP Sensitization Outside the Mediterranean Area

The first food allergen from the LTP family described was Pru p 3 (initially designated Pru p 1) from peach [4, 5]. Up to now, numerous LTPs have been reported and characterized as food allergens, and respiratory LTP allergens have been found in tree and weed pollen as well as food (e.g., wheat LTP contributing to baker’s asthma), but also as contact allergens. However, almost all reports are from studies performed in Southern Europe countries of Southern European countries indicating the important clinical relevance of this allergen family in that area (reviewed in [2]). In the Mediterranean basin, more than 90% of patients with reactions to plant-derived foods, especially fruits from the *Rosaceae* family, are sensitized against the respective LTPs, and almost all peach allergic patients with severe systemic reactions show sensitization to Pru p 3 [6], the clinically most important and best characterized food LTP. In line with this, reports on LTP-mediated allergies were initially limited exclusively to the Mediterranean area. In contrast, nowadays, there is an increasing awareness that sensitization to LTPs is not exclusively limited to Southern Europe (reviewed in [7]). Meanwhile, sensitization to LTPs outside the Mediterranean area has been documented by several case reports and studies applying component-resolved diagnosis (CRD) (Table 1). However, the pathogenesis and clinical significance of LTP allergies for these patient populations is controversially discussed and not fully characterized.

**Table 1:** Epidemiology of LTP sensitization in the non-Mediterranean area (exemplary reports)

LTP	Source	Country	Prevalence		Patients (inclusion criteria)	Assay	Ref
			%	*N*			
**Pru p 3**	**Peach**	CN (Northern)	**96**	23/24	Peach allergy with mugwort allergy (history): all Artv3+	CAP	[8•]
					Peach allergy without mugwort allergy (history): 6/15 Artv3+ and mugwort allergy without peach allergy (history): 12/31 Artv3+		
		ES (Northern)	**88–96**^**1**^	63–69^1^/72	Adults with plant food allergy (history, Prup3 SPT+, birch pollen and profilin neg.)	^1^ISAC/CAP	[9]
		AT	77	10/13	Plant food allergy (history of anaphylactic reaction or SPT+ > 8 mm)	ISAC or CAP	[10]
		CN	80	86/107	Mugwort pollen-related food allergy (history): 69% Arah9+, 63% Cora8+	CAP	[11•]
**Cor a 8**	**Hazelnut**	NL	**100**	8/8	Pediatric hazelnut allergies (spec. IgE, DBPCFC+)	CRD (RAST, IB)	[12]
			6	1/18	Pediatric hazelnut patients (spec. IgE, without objective symptoms upon DBPCFC)		
		DK	5	1/20	Adult hazelnut allergies (DBPCFC+)	CRD (CAP)	[13]
		CH	15	3/20			
		NL	8	3/40	Pediatric hazelnut allergies (DBPCFC+)	CRD (CAP)	[14]
			5	2/39	Adult hazelnut allergies (15 DBPCFC+ & 24 history)		
		NL	12	5/42	Hazelnut and/or apple allergies (history, HN and/or apple spec. IgE or SPT+), 9/42 Prup3+, 3/9 anaphylactic HN patients were Cora8+	CAP	[15]
		Central/Northern Europe	< 15	< 51/343	Hazelnut allergies (history, with & w/o DBPCFC), 343 from 8 EU cities (non-Mediterranean)	CRD (CAP)	[16]
		CN	63	59/107	Mugwort pollen-related food allergy (history): 69% Arah9+, 80% Prup3+	CAP	[11•]
**Ara h 9**	**Peanut**	US	**67**	4/6	Adult PN allergies (history, PN spec. IgE), 3/4 Arah9+ US patients were Prup3+	CAP, IB^2^	[17]
		DE	6	2/35^2^			
		SE	14	5/35	Pediatric PN allergies (food challenge positive or history, PN spec. IgE)	CRD (CAP)	[18]
		US	8	2/30			
		JP	15^3^–**58**^4^	4^3^–15^4^/26	Pediatric PN allergic (OFC+), ^3^ > 0.35 kU/L, ^4^ > 0.1 kU/L, ^3,4^ not significant vs tolerant group	CRD (CAP)	[19]
		SE	16^5^	4^5^/25	Pediatric PN allergies (DBPCFC+, PN spec. IgE or SPT+), ^5^not significant vs tolerant group	CRD	[20]
		TW	24	7/29	Preschool children (history, preselected by PN spec. IgE ≥ 3.5 kU/L = CAP class 3)	CD (CAP)	[21]
		UK	20	38/192	Pediatric PN allergies (history), subgroup (*n* = 2) Arah9+ but Prup3 negative	CRD (CAP)	[22]
		TH	26	5/19	Pediatric/adolescent PN allergic (history or DBPCFC+, PN spec. IgE), 1 Arah9+/21 PN tolerance	CRD (CAP)	[23]
		Europe	24	14/59	Children and adults (40 history or 28 DBPCFC+), 59 from 8 non-Mediterranean EU countries (Arah9+ in CH, NL, UK, CZ)	CRD (CAP)	[24]
		CN	**83**	15/18	38 Adult/adolescent PN sensitized patients: 18 symptomatic and 20 tolerant, 14 Arah9+/20 PN tolerant	CRD (CAP)	[25]
		UK	63	22/35	Patients with pollen-food syndrome, preselected by LTP-allergy (Prup3+): 52% Cora8+, 86% Jugr3+, 83% Prup3+, 23% Tria14+, 60% Artv3+, 17% Olee7+, 66% Plaa3+	ISAC	[26]
		CN	**69**	64/107	Mugwort pollen-related food allergy (history): 80% Prup3+, 63% Cora8+	CAP	[11•]
**Mal d 3**	**Apple**	NL	1	1/99	Adult apple allergy (history, apple SPT+)	CRD (RAST)	[27]
		AT	2	2/94			
		PL	95	20/21	Pediatrics with birch pollen and apple allergy (birch and apple spec. IgE), putative LTP in apple extracts by IB (data from individual sera not shown)	CAP, IB	[28]
		DE	Case report	*n* = 1	Adult with FDEIA to apple (history, apple spec. IgE)	CAP	[29]
**Tri a 14**	**Wheat**	DE	3	1/40	Patients with baker’s asthma (history, wheat flour spec. IgE)	CAP	[30]
**Act d 10**	**Kiwi**	IS	3	1/29	Children and adults (history), 266 from 9 non-Mediterranean EU countries	CRD (CAP)	[31]
		Eastern Europe	9	5/56			
		Western/Central Europe	11	20/181			
**Api g 6**	**Celery**	AT	38	12/32	Celery allergies (celeriac spec. IgE and/or SPT+), no correlation od Apig6 with Apig2 or Artv3	ELISA	[32]
**Len c 3**	**Lentil**	AT	Case report	2/3	Adult lentil or legume allergy (history, lentil spec. IgE), all Prup3+	CAP, IB	[33]
**Pru av 3**	**Cherry**	DE	3	3/101	Adult cherry allergies (history, cherry spec. IgE)	EAST	[34]
		CH	4	1/24	Adult cherry allergies (history, DBPCFC+, 23/24 cherry SPT+)	CRD (SPT)	[35]
		DE & CH	5	1/22	Adult cherry allergies (DBPCFC+, 20 CH and 2 DE)	CRD (CAP)	[36]
			12	12/99	Adult (history, 97 CH & 2 DE)		
**Vit v 1**	**Grape**	DE	Case report	*n* = 1	Adult case history (spec. IgE and SPT+)		[37]
**Vac m 3**	**Blueberry**	DE	Case report	*n* = 1	Adult, case history (spec. IgE and SPT+)		[38]
**Art v 3**	**Mugwort**	AT & CA	**89**	8/9	Mugwort (9) and/or ragweed (10) allergies (history, SPT+, spec. IgE), 10 AT and 9 CA	CRD (array, ELISA)	[39]
		CN (Northern)	**100**	24/24	Mugwort allergies with peach allergy (history): 23/24 Prup3+	CAP	[8•]
			39	12/31	Mugwort allergy without peach allergy (history): 9/31 Prup3+, all 9 also Artv3+		
		CN	**53**	127/240	Mugwort allergy (history, spec. IgE)	CRD (CAP)	[40••]
		(Southwest)	9	3/32	In Yunnan		
		(Northern)	**66**	117/178	In Shanxi (but 25%, 7/30 in Shandong with lower mugwort pollen load)		
		**CN**	**73**	108/148	Mugwort allergies from Peking (with/without food allergy) (history, mugwort SPT+ and spec. IgE)	CAP	[11•]
			**87**	93/107	Mugwort allergic with food allergy		
			37	11/31	Mugwort allergic without food allergy		
		CN	**57**	36/63	Patient with autumn (incl. mugwort 94%) pollinosis (history, pollen SPT+ and spec. IgE) from Peking	CAP	[41]
			**79**	26/33	Patients with autumn pollinosis and food allergy (history, SPT+ and/or spec. IgE), 52%		
			33	10/30	Patients with autumn pollinosis w/o food allergy, 48%		
**Can s 3**	**Cannabis**	BE	**72**	18/25	Cannabis allergies (history) with likely anaphylactic reactions (25/120), 92% of Cans3+ also LTP+	CAP, BAT	[42]
**Jug r 3**	**Walnut**	CH	42	13/31	Adolescent/adult walnut allergic patients (history or DBFCFC+ or OFC+)	CRD (CAP)	[43•]
		DE	32	10/31			
**Amb a 6**	**Ragweed**	AT & CA	30	3/10	Mugwort (9) and/or ragweed (10) allergies (history, SPT+, spec. IgE), 10 AT and 9 CA	CRD (array, ELISA)	[39]

*CN* China, *ES* Spain, *AT* Austria, *DK* Demark, *NL* Netherlands, *US* United States, *DE* Germany, *UK* United Kingdom, *SE* Sweden, *TW* Taiwan, *JP* Japan, *TH* Thailand, *PL* Poland, *IS* Iceland, *BE* Belgium, *CZ* Czech, *CA* Canada, *DBPCFC+* double-blind placebo-controlled food challenge positive, *OFC+* open food challenge positive, *SPT+* skin prick test positive, *PN* peanut, *HN* hazelnut; Bold: LTPs classified as major allergens (≥ 50% prevalence in the respective patient group)

On the one hand, the importance of pollen and food LTP in the sensitization process outside the Mediterranean area has been demonstrated. The LTP syndrome in mugwort and/or peach allergics from Northern China can either be driven by the mugwort pollen LTP Art v 3 (see chapter 3) or Pru p 3 which was identified as major allergen in peach allergics tolerating mugwort [8•]. Pru p 3 is suggested even as a marker allergen for LTP sensitization in the non-Mediterranean area [9]. Furthermore, the prominent role of Pru p 3 is supported by a study showing sensitization to Pru p 3 in patients with allergy to raspberry and apricot from Austria [10]. However, these patients were preselected by a history of severe allergic symptoms. Sensitization to cannabis LTP Can s 3 was reported in Belgium among cannabis allergic patients, and with a frequency of 72% reporting severe reactions [42]. Remarkably, Can s 3 positive patients had high prevalence (92%) of sensitization to other LTP such as apple Mal d 3, hazelnut Cor a 8, walnut Jug r 3, wheat Tri a 14, and mugwort Art v 3 but also Pru p 3. In addition, Anantharachagan et al. reported eight patients with LTP-driven allergy in Northwestern England [44]. Patients were sensitized to a broad panel of LTPs, at which 7/8 were reactive to Pru p 3 and 5/8 were reactive to Ara h 9. Data from these reports suggest that outside the Mediterranean area both Pru p 3 and Art v 3 can act as immuno-dominant LTP.

On the other hand, in the majority of studies from the non-Mediterranean area, sensitization to LTPs seems to be of limited importance, and LTPs were frequently classified as minor allergens. Case reports of allergic reaction to grape LTP Vit v 1 in a German wine maker [37] and suspected allergy to Vit v 1 and apple LTP Mal d 3 in a 12-year-old female from Australia [45] are available. In addition, other case reports suggested the association of a LTP sensitization and food-dependent exercise-induced anaphylaxis (FDEIA) in a patient from Poland after eating several, also LTP-containing, foods [46] and demonstrated the presence of IgE to apple LTP in a German patient with food allergy to apple but without sensitization to Bet v 1-like proteins, storage proteins, or profilin [29]. Of note, sensitization against LTPs can also cause occupational allergies outside the Mediterranean area [30]. However, the reactivity to wheat LTP Tri a 14 was a less frequent trigger (2.5%) of baker’s asthma in Central Europe [30] than in Southern Europe (60%) [47]. In a retrospective explorative study, 15% of 305 adult patients visiting the outpatient clinic in Utrecht (the Netherlands) were sensitized to food LTPs, as measured by the ISAC112 microarray methodology. The majority of LTP-positive patients was co-sensitized to PR-10 allergens rather than to storage proteins, and only a minority of subjects was mono-sensitized to LTPs [48]. An additional survey performed in Belgium ruled out a high percentage of more than 25% out of 718 patients with pollen and/or food allergy be sensitized to any of the tested LTPs [49, 50]. Using a panel of four foods and two pollen LTPs, the study demonstrated IgE reactivity not to be correlated with a clinical phenotype due to frequent clinically insignificant sensitization. However, the authors did not provide an explanation for the high frequency of sensitization to LTP in this study cohort.

So far, several CRD studies contribute to the understanding of the role of LTPs outside the Mediterranean area. One of the first studies applying LTP in CRD was performed in cherry allergic patients from Spain and Switzerland [35]. Ballmer-Weber et al. [35] found that only 1 out of 24 double-blind placebo-controlled food challenge (DBPCFC)-positive Swiss cherry allergic patients was sensitized to cherry LTP Pru av. 3, which was classified as a major allergen in Spain (prevalence of 89%). A follow-up study with patients from Germany and Switzerland revealed that only 11% (13/121) of cherry allergics were sensitized to Pru av. 3 [36]. Remarkably, all study subjects reported exclusively OAS, and only 1/11 patients tested was mono-sensitized to Pru av. 3. Similar results were obtained for Dutch and Austrian patients (*n* = 193) selected by history of adverse reactions and positive SPT to apple at which sensitization to Mal d 3 was almost not observed (< 3%) [27]. CRD of kiwi fruit allergy across Europe revealed a frequency of sensitization to kiwi LTP Act d 10 of 3–11% outside the Mediterranean area vs 22% in Southern Europe [31]. An early study in a birch-endemic area in the Netherlands revealed that sensitization to purified hazelnut LTP Cor a 8 was associated with objective symptoms in all children with IgE to hazelnut LTP [12]. Interestingly, 6/8 tested sera did not react to Pru p 3. Using the ISCAC microarray approach [51], an age-dependent association of systemic reactions to hazelnut with sensitization to Cor a 8 was found in 12%, 17%, and 33% of pre-school, school-aged children, and adults from Belgium, respectively, but not in patients reporting OAS. Later studies did not confirm the suggested prominent role of Cor a 8 in systemic allergic reactions to hazelnut, suggesting that likely other inclusion criteria were applied or by false-positive results due to potential contamination of natural Cor a 8 by, e.g., seed storage proteins [13, 14, 52]. A CRD study of hazelnut allergy across Europe in DBPFC-positive patients revealed a prevalence of sensitization to Cor a 8 of 5% (1/20) in Denmark and 15% (3/20) in Switzerland [13], with only 1/4 of these Cor a 8-sensitized hazelnut allergic patients reporting severe reactions. In the control group consisting of birch pollen allergics with tolerance to hazelnut, no IgE binding to Cor a 8 was detected. Unfortunately, this study did not explore further individual sensitization patterns, such as to Pru p 3 or pollen LTPs. A Dutch study by Masthoff et al. [14] reported no substantial difference of Cor a 8 sensitization rates of 5% and 8% in children and adults with objective symptoms to hazelnut, respectively. In addition, a so-called molecular map of hazelnut allergy across 12 European cities revealed Cor a 8 sensitization of minor importance in almost all cities by a frequency of less than 15% (except Madrid and Athens) [16]. Similar results were obtained from the analysis of the sensitization pattern in hazelnut-positive individuals across the USA showing that approximately 10% were sensitized to Cor a 8 regardless of the age, and Cor a 8 sensitization was considered not a predictive marker for severe reactions [53]. Cor a 8 was not a reliable diagnostic marker in hazelnut open food challenge (OFC)-positive children from Japan [54]. Moreover, the low prevalence of sensitization to Pru p 3 in Japanese children was attributed to eating habits, since peaches are consumed without peel in Japan [55]. Therefore, LTPs that preferentially accumulate in the peel are almost removed. In terms of the sensitization pattern to peanut, a previous study showed a heterogeneous reactivity to peanut LTP Ara h 9 in different populations investigated: IgE reactivity was found in 29/32 Spanish and 6/41 non-Mediterranean peanut allergics [17]. Similar results were published by Vareda et al. [18]: Ara h 9 sensitization was confirmed in 8% and 14% of peanut allergics in the USA and Sweden vs 60% in Spain. Of note, the highest percentage of mono-sensitization to Ara h 9 (but no IgE binding to other peanut allergens) was found in patients from Spain (18/50), which corresponded to systemic reactions in 16/18 patients. Later, IgE sensitization pattern in peanut allergy was investigated within the EuroPrevall study [24]. Briefly, 68 peanut allergic subjects from 11 European countries, thereof 59 patients from 8 countries not belonging to the Mediterranean area, were enrolled. Approximately 20% (12/59) of these patients were sensitized to Ara h 9. Sensitization to Ara h 9 (and to Bet v 1-like protein Ara h 8), but not sensitization to storage proteins, was frequently associated with tolerance to peanuts. However, in contrast to Vereda et al. [18], the authors [24] further concluded that sensitization to Ara h 9 usually seems not to be acquired in childhood. Moreover, sensitization to Ara h 9 in 3/33 Swedish patients, all co-sensitized to Bet v 1, was not associated with severe clinical reactions [56]. Recently, interesting data were provided by a Chinese CRD study reporting that the most common allergen in peanut-sensitized subjects is Ara h 9 (in 83% of 38 subjects), of which more than half (*n* = 24) suffered from mugwort pollinosis and peach allergy [25]. In 18/38 subjects, peanut sensitization was symptomatic, and 15/18 peanut allergic patients were reactive to Ara h 9, among whom 12 were Ara h 9 mono-sensitized. Of the 5 patients presenting severe reactions, 4 were mono-sensitized to Ara h 9 [25]. CRD in walnut allergic patients from Spain, Switzerland, and Germany showed that a high rate of 32% and 42% of patients from Germany and Switzerland were sensitized to walnut LTP Jug r 3, respectively [43•]. Based on all three patient groups, sensitization to Jug r 3 was not significantly associated with severe symptoms. Remarkably, in patients from Switzerland and Germany, reporting systemic reactions, the frequency of reactivity to Jug r 3 was moderate (15/32), but none of the patients was mono-sensitized to Jug r 3. Severe reactions were reported by patients sensitized to storage proteins, but were not associated with LTP or PR-10 allergens. In contrast, all patients from Spain with severe symptoms exhibited IgE to Jug r 3, and 5/8 patients were Jug r 3 mono-sensitized (considering six walnut allergens tested). Data further suggest that sensitization to LTPs outside the Mediterranean basin is accompanied by a broad reactivity to other allergens from the same source which in case of the storage proteins contribute to more severe reaction rather than LTP.

In summary, sensitization patterns to LTP in China and Central/Northern Europe do not resemble the Mediterranean serotype. The prevalence of LTP sensitization is not comparable, and lower in patients recruited outside the Mediterranean area (except China), defining them as minor allergens. Typically, patients are frequently poly-sensitized to broad spectrum of other allergens from the same allergenic source. In line with this, diagnosis of LTP sensitization using plant extracts is limited due to frequent concomitant pollen allergies, e.g., to birch pollen, and cross-reactive allergens thereof, but can be dissected by CRD. In general (except China), LTP reactivity seems to be of less clinical importance when patients are co-sensitized to other allergic proteins. It needs be taken into account that heterogeneous results in terms of the epidemiology of LTP-mediated allergies can be confounded by non-standardized diagnostic procedures or by different inclusion criteria of the studies. The explanation for the different geographical relevance of LTPs remains elusive since food and vegetables consumed in Central and Northern Europe express high levels of LTPs, and different dietary habits and food processing practices may not serve as solely explanation. In this context, a potentially different pollen environment with different exposure of LTP-containing pollen needs to be taken into consideration.

## Potential Role of LTP in Pollen-Related Food Allergies

One of the key questions is whether the pathogenesis of the LTP syndrome also differs between geographical areas: which pollen and/or plant food LTPs outside the Mediterranean area can act as genuine primary sensitizers?

Evaluating studies from Southern Europe, it has already been suggested that besides the primary sensitization against Pru p 3 and subsequent cross-reactivities with homologous LTPs from pollen and food, it might also be possible that pollen LTPs can induce sensitization leading to a subsequent cross-reactivity with food LTPs, at least in some patients [57]. Sensitization to pollen is the most dominant factor to trigger sensitization and allergy to plant foods [1, 58]. Therefore, geographical differences in the pollen exposure across Europe have been discussed to explain differences in food allergy. In line with this, the LTP syndrome was suggested to be caused by exposition towards pollen LTPs with greater distribution in Southern Europe [59]. It is tempting to speculate that the geographically different abundance of allergenic LTP-containing pollen can be associated with the geographically different prevalence of LTP-mediated food allergies. So far, according the IUIS allergen nomenclature database, allergenic pollen LTPs have been identified from short ragweed (*Ambrosia*, Amb a 6), mugwort (*Artemesia,* Art v 3), thale cress (*Arabidopsis,* Ara t 3), field mustard (*Brassica,* Bra r 3), olive tree (*Olea,* Ole e 7), and plane tree (*Platanus,* Pla a 3, Pla or 3), as well as from pellitory (*Parietaria*, Par j 1/2, Par m 1, Par o 1). Remarkably, the exposure to these pollens is not restricted to the Mediterranean area, but pollen counts and peak pollen exposure may vary between regions. Although correlation between sensitization to certain LTPs from pollen and from foods has been described, the role of pollen- and food-derived LTPs as a primary sensitizer largely remains elusive.

### Southern Europe

Mugwort (*Artemesia*) pollen-related peach allergies and the engagement of LTPs have been initially reported from Italy [60, 61] and Spain [62–65]. However, the impact of respiratory exposure towards pollen LTPs (especially Art v 3 from mugwort and Pla a 3 from plane tree) on the development of food allergies is controversially discussed in Southern Europe. Some authors describe LTP-mediated respiratory allergies as a consequence of a primary sensitization against Pru p 3 with subsequent IgE cross-reactivity with Art v 3, but frequently without clinical manifestation of pollinosis [60, 65]. No or low IgE reactivity to mugwort was determined in Italian peach allergic patients, mono-sensitized to LTPs. The authors concluded that mugwort was unlikely to represent the primary sensitizer and that this scenario might be a geographically restricted phenomenon [61]. In contrast, another study showed that Pru p 3 was not able to inhibit IgE binding to Art v 3—but vice versa—and the authors postulated pollen Art v 3 to have primary sensitizing properties and to trigger LTP-mediated food allergy [64]. The authors speculate that exposure to peach and mugwort or plane tree pollen in areas where birch pollen is not abundant could lead to LTP allergy [27]. The possibility of pollen LTP to act as primary sensitizer was further supported by Lauer et al. [66], showing IgE binding to Pla a 3 in some Spanish patients without co-sensitization to Pru p 3. In terms of the cross-reactive properties, Salcedo et al. [67] classified pollen LTPs into two groups. The first group referrs to, LTPs with less than 35% aa-sequence identity and no cross-reactivity with Pru p 3, e.g. ragweed Amb a 6, olive Ole e 7 and pellitory pollen Par j 1/2. The second group referrs to LTPs with with more than 45% aa-sequence identity to Pru p 3, e.g. mugwort Art v 3 and plane tree pollen Pla a 3, resulting in plant-food pollen cross-reactivity. Gadermaier et al. demonstrated cross-reactivity of LTP Api g 2 from celery stalk with mugwort pollen Art v 3 and Pru p 3 in Italian patients and suggested that food LTP sensitization may also be mediated by pollen LTP [68]. Results were supported by Scala et al. [69] showing that Pla a 3 and Art v 3-mediated respiratory symptoms were associated with sensitization to food LTPs in Italian LTP-positive subjects, and mugwort pollen extract was capable to inhibit the IgE binding to food LTPs up to 50–100%. Interestingly, 63% of Pru p 3-negative-tested patients (representing 18% of all LTP-positive subjects tested) were positive to Art v 3/and or Pla a 3, and patients with sensitization to Art v 3 or Pla a 3 were frequently mono-sensitized, not recognizing other allergens from mugwort or plane pollen [69]. Vice versa, only a small number of patients displayed co-sensitization to birch pollen–derived allergens and those that did reported mild symptoms. Finally, Sanchez-Lopez et al. [65] reported sensitization to Pru p 3 and Art v 3 to be frequently associated and suggested two pathomechanisms, either respiratory allergy induced by pollen Art v 3 in Art v 3 mono-sensitized subjects or by cross-reactivity after primary Pru p 3 allergy and exposure to mugwort Art v 3.

### Non-Mediterranean Area

Recent data provide further evidence for an important role of LTPs in the mugwort-peach syndrome outside the Mediterranean area, in particular in China. Respiratory allergy to mugwort pollen causing asthma is a well-known phenomenon in particular in Northern China where the exposure to mugwort is very high [8•, 40••, 70••]. The LTP Art v 3 from mugwort pollen was identified as a predominant allergen in Chinese mugwort allergic patients with associated peach allergy [70••]. Remarkably, it has been suggested that high exposure to mugwort pollen in Northern China can lead to primary sensitization against Art v 3, which promotes the subsequent development of peach allergy by IgE cross-reactivity with Pru p 3 in some patients [8•, 70••]. All 24 patients with mugwort and peach allergy were sensitized to Art v 3, and 23 patients were sensitized to Pru p 3 [8•]. In almost all patients, specific IgE levels to Pru p 3 were lower than to Art v 3. In contrast, in a group of peach allergic patients without symptoms to mugwort, IgE responses to Art v 3 were lower than to Pru p 3. In addition, in mugwort allergic patients who tolerate peach, 12/31 patients had specific IgE to Art v 3, and 9 out of these 12 patients displayed a less prominent and clinically insignificant IgE reactivity to Pru p 3. Furthermore, in patients with prominent Art v 3 immune response, Pru p 3 was not able to compete IgE binding to Art v 3, indicating the existence of Art v 3-specific IgE epitopes. The data suggest Art v 3 to act as a primary sensitizer and provide convincing support for a prominent role of Art v 3 in the pathogenesis of mugwort allergy and the translation into Pru p 3-mediated peach allergy in a subgroup of patients [8•]. In China, allergic reaction to mugwort pollen is strongly associated with peanut allergy. Of note, Ara h 9-positive peanut allergics as well as Ara h 9-positive but peanut-tolerant patients, both with mugwort pollinosis, showed higher specific IgE values to Art v 3 than to Ara h 9 [25]. Vice versa, peanut allergics without mugwort pollinosis showed a strong correlation with peach allergy and higher Pru p 3-specific IgE than Ara h levels. Finally, the data suggest that either Art v 3 or Pru p 3 rather than Ara h 9 are important LTPs in China which can trigger secondary peanut allergy. However, the role of LTPs in the mugwort pollen-related food allergy was further explored in recent studies. Gao et al. showed by CRD that mugwort pollen allergic patients from Northern China more likely have severe symptoms and being sensitized to a cluster of at least three mugwort allergens including Art v 3 rather than sensitized to one or two allergens [40••]. The frequency of sensitization to Art v 3 was 53% in mugwort pollen allergic patients from overall China, with a highest prevalence of 66% and highest Art v 3-specific IgE values in Shanxi, Northern China. Overall data from China [8•] are in agreement with Lombardero et al. [64], showing that Pru p 3 was not able to inhibit IgE binding to Art v 3 but vice versa. Another study including 148 mugwort allergic patients, of which 107 patients reported an associated food allergy, aimed to correlate the IgE-level of LTP with the severity of allergic reactions [11•]. Interestingly, 73% of the mugwort allergic patients were sensitized to Art v 3, and peach accounted for the highest number (64%) of reactions to food. In line with this, food allergy correlated with high frequency of IgE to Art v 3 (87% compared to only 37% in food tolerant patients) and with sensitization to Pru p 3 (80%), Ara h 9 (69%), and Cor a 8 (63%). Food-induced systemic reactions were associated with higher specific IgE levels against LTPs, along with a prominent response to Pru p 3 (prevalence of 100%) in comparison to patients with OAS (50%). In summary, the study showed a strong involvement of Pru p 3 in the manifestation of (severe) allergic symptoms and provides further evidence for a potential role of Art v 3 as a sensitizing agent. However, it would have been interesting to compare specific IgE levels for Art v 3 and Pru p 3 in mugwort-peach allergics with those in peach allergics but mugwort-tolerant patients as well. It still remains unclear whether there are cases where food allergy was developed after the establishment of pollinosis. Most recently, further support for pollen-driven LTP sensitization came from by an independent study from China [41], showing a high prevalence of sensitization to Art v 3 in patients with pollen-related food allergy rather than without food allergy. The authors reported only a moderate correlation (*R* = 0.53) between Art v 3- and Pru p 3-specific IgE levels. The presence of IgE to Art v 3 has been proposed as a predictive biomarker for season-specific pollen-related food allergy.

Considering the role of LTPs in the pollen-food syndrome in non-Mediterranean countries from Europe, the data are heterogeneous. Taking into account the sensitization pattern to hazelnut allergens across Europe, Datema et al. [16] did not find a substantial correlation of hazelnut LTP Cor a 8 sensitization with that to mugwort or plane pollen. However, the authors discussed the potential engagement of LTPs from pollen and food as a source of primary sensitization in Southern Europe. Conclusive data are limited since the exact prevalence of food allergy in patients with mugwort pollinosis has not been determined in Europe [71]. Applying the ISAC allergen microchip system, another study found no difference between LTP allergics from the UK (selected by Pru p 3 sensitization) and Italy in terms of the sensitization pattern to individual LTPs, including Ara h 9, Cor a 8, Jug r 3, Tri a 14, Art v 3, Pla a 3, and Ole e 7 [26]. Here, a high rate of co-sensitization to Art v 3 (60%/81%) and Pla a 3 (66%/84%), but not to Par j 2, was determined in both groups. However, correlation between Pru p 3 and Art v 3 in LTP allergics from the UK was found to be higher than reported by Faber et al. [50] in Belgian patients (*r* = 0.823 vs *r* = 0.48). Remarkably, Pla a 3 was only positive in patients sensitized to Pru p 3 but not in the group with pollen-food syndrome. Data from this study support the view that Pla a 3 does not act as a primary sensitizer in the study population [26].

Like for Art v 3, a role as primary sensitizer of olive pollen LTP Ole e 7 leading to subsequent peach LTP sensitization was recently suggested for patients from areas in Spain with high exposure to olive pollen (5000 grains/m^3^) [72•]. The study reported 13 Ole e 7 mono-sensitized patients with limited IgE cross-reactivity to Pru p 3. An independent co-sensitization to both LTPs may point to primary sensitizing properties of both allergens. The role of Ole e 7 in other areas where olive trees are abundant (Australia, South America and California/USA, China, India) is still elusive.

In summary, until now, there are no concise reports of the clinical role of pellitory, plane, or olive pollen in the manifestation of the LTP-food syndrome. More importantly, unlike LTP-mediated food allergy in Southern Europe, which in the majority of cases occurs independent of pollen hypersensitivity, LTP-mediated food allergy in China seems to mainly originate from sensitization to mugwort pollen LTP. However, the reason for this divergent role of LTPs is unclear. On the one hand, pollen LTP-driven food allergies should be of clinical relevance even in the Mediterranean area; on the other hand, food-derived LTPs should act in a similar way as genuine allergens even outside the Mediterranean area. However, the mugwort pollen counts in China are considered to be substantially higher than in Southern Europe, and according to cross-inhibition experiments, Art v 3 likely comprises most of the IgE-binding epitopes of the food LTPs. Despite an initial study showing the predominant T cell reactivity of Pru p 3 in comparison to Cor a 8 [73], the role of LTP-derived T cell epitopes needs to be further elucidated in order to explain the phenomenon of cross-sensitization in different allergic populations. Finally, experimental models are required showing that pollen LTP sensitization facilitates food LTP-mediated allergies.

## Clinical Manifestation of LTP-Mediated Food Allergies in Poly-sensitized Patients

The sensitization pattern to allergens strongly depends on the geographical area, including environmental conditions and exposure to respiratory and food allergens. Although sensitization to grass and olive pollen is a known phenomenon in certain regions in Spain, patients with LTP allergies in the Mediterranean basin are frequently mono-sensitized to food LTPs. In contrast, sensitization to LTPs in patients in Central and Northern Europe frequently is accompanied by co-sensitization to birch pollen allergens characterized by cross-reactive properties with homologous food allergens. In line with this, precise diagnosis of food allergy is often hampered by extract-based assays, whereas CRD allows to correlate the reactivity of individual allergens with clinical manifestation.

### Southern Europe

One of the first studies suggesting that a high load of birch pollen might decrease the probability of primary sensitization to LTP was by Fernandez-Rivas et al. [27]. The authors reported that sensitization to Bet v 1 reduces the risk of an IgE response to apple LTP Mal d 3 by a factor of 3.5. However, a sensitization to mugwort and plane tree pollen was strongly associated with IgE to Mal d 3 [27], likely due to the presence of cross-reactive LTPs in these pollens. Of note, in Spain, 91% (20/22) of cherry allergic patients were sensitized against LTP Pru av. 3, but only two patients were sensitized against other known cherry allergens, and 50% of patients reported systemic reactions after consumption of cherries [36]. Moreover, the reactivity to Pru p 3 correlated with severe reactions, whereas co-sensitization to profilin was associated with OAS [74]. This observation was confirmed by a study showing that patients with Pru p 3-specific IgE are less likely to develop severe symptoms when they are poly-sensitized by IgE reactivity to profilin Pru p 4 and Bet v 1-like protein Pru p 1 [75]. Moreover, two different sensitization patterns of apple allergic patients were described in Spain: Patients were either mono-sensitized to apple LTP Mal d 3 and Pru p 3 or co-sensitized to apple profilin (or Mal d 1) [76]. Here, LTP mono-sensitized patients showed higher incidence of generalized symptoms and OAS in comparison to profilin co-sensitized patients. The data are supported by a study showing that the IgE reactivity to LTP was correlated with a lower frequency of severe reactions when the patients were co-sensitized to profilin [77] or both profilin and Bet v 1-like protein [69]. In addition, the latter study [69] demonstrated that subjects who reacted to > 5 LTPs report a higher frequency of food-induced systemic reactions [69]. Although the phenomenon of LTP allergy and co-sensitization to profilin and Bet v 1-like allergens has been described for a subgroup of patients from the Mediterranean area, the sensitization pattern resembles the situation frequently found for patients in birch pollen endemic areas of Central and Northern Europe.

### Non-Mediterranean Area

Until now, only a couple of European CRD studies that have included patients from outside the Mediterranean area were aiming to correlate sensitization patterns with clinical reactivity to LTPs (see above). Unfortunately, however, in most studies, the sensitization pattern of individual patients was not presented.

In a study conducted in Belgium, 718 patients with history of inhalant and/or food allergy were monitored for LTP and Bet v 1 reactivity [50]. A high rate of asymptomatic IgE reactivity to LTP was explained by a high frequency of patients (79%) co-sensitized to Bet v 1 [50]. In this study, the Basophil Activation Test (BAT) was a demonstrated as a suitable method to dissect LTP-positive patients with clinically significant sensitization from those with asymptomatic sensitization: Pru p 3 failed to induce basophil activation in Pru p 3-positive patients that tolerated peach. Noteworthy, all Pru p 3-positive patients with OAS to peach were sensitized to Bet v 1. Recently, LTP sensitization has been reported as a risk factor for severe allergic symptoms in Central Europe, and Pru p 3 was suggested as a prototypic marker allergen for LTP sensitization even in the patients from Central Europe [10]. Here, 10/13 patients from Austria, preselected by history of severe reactions to food or strong SPT reaction, were sensitized to Pru p 3, and 6/10 Pru p 3-positive patients exhibited co-sensitization to major birch pollen allergen Bet v 1. The data are contradicting other studies showing that co-sensitization to birch allergens is related to less severe reactions. However, it needs to be mentioned that patients with severe reactions were preselected in the present study. The effect of pollinosis on the geographical pattern of LTP-food allergies was discussed by Rial et al. [78]. The authors pointed out that (1) the hypersensitivity reactions to food LTP in birch-endemic areas appear to exist, but (2) high levels of birch pollen are likely to protect from LTP-mediated allergy. The authors refer to the manifestation of LTP-food allergies by (1) primary sensitization to food LTP in the absence of pollinosis, (2) primary sensitization to pollen LTP and secondary food allergy, or (3) independent sensitization (co-sensitization) to LTP from pollen and food as suggested previously by Zuidmeer et al. [57]. A recent study by Decuyper et al. was aimed at comparing molecular and clinical characteristics of LTP sensitization with and without co-sensitization to pollen in patients recruited from the Mediterranean area and Central Europe [79]. Depending on the patient group, the authors concluded that different diagnostic test parameters (either Mal d 3-specific IgG4/IgE and BAT, or Pru p 3-specific IgE/total IgE) would be of added value to identify clinically relevant sensitization. Moreover, it appeared that symptomatic patients with Pru p 3 and/or Mal d 3 sensitization in Spain reacted to lower allergen concentrations than in Belgium. This finding was attributed to the fact that patients from Belgium were more frequently poly-sensitized to pollen.

**Fig. 1:**
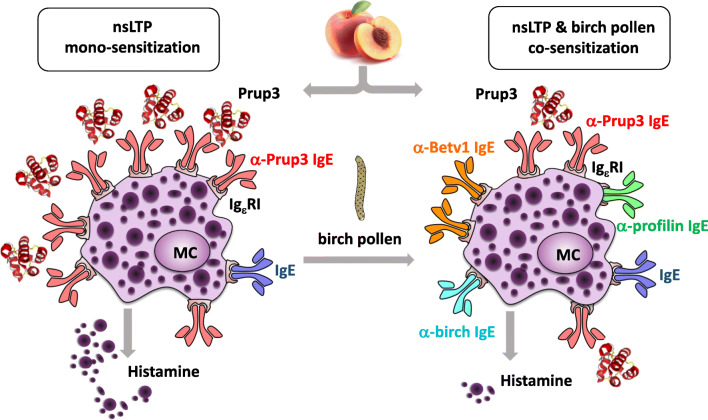
Model of Pru p 3 induced mast cell (MC) activation in patients with LTP mono-sensitization (left) and patients co-sensitized to unrelated allergens (right), e.g., from birch pollen or the same source (profilin and Bet v 1-like protein). Due to the high density of LTP-specific IgE on the surface of MC in LTP mono-sensitized patients, Pru p 3 efficiently induces mediator release which corresponds with a higher probability of severe reactions

In summary, mono-sensitization to LTPs seems to correlate with a stronger clinical reactivity. A possible mechanism for this observation could be that IgE receptors on the effector cells are primarily occupied by LTP-specific IgE, which likely facilitates efficient allergen-induced crosslinking of the IgE-receptor and subsequent effector cell activation (Fig. 1). In contrast, outside the Mediterranean area, the presence of LTP-specific IgE usually is accompanied by the expression of IgE directed against other allergens, in particular from birch pollen. The co-sensitization to unrelated allergens, e.g., Bet v 1 or pollen profilins, either derived from the same source or from different species, is suggested to mediate a “protective” effect on the manifestation of an LTP allergy [78]. However, we have to consider that the ratio of LTP-specific IgE and total IgE might also affect cell activation and clinical reactivity.

## Conclusions

Although LTP-mediated allergies are common in the Mediterranean area, the number of reports on allergies caused by LTPs outside this area is continuously increasing and strengthens the awareness that the LTP syndrome is not restricted exclusively to Southern Europe. The reason probably is not an increasing incidence of LTP allergies rather than the utilization of purified allergens for CRD allowing to dissect sensitization to LTP from other allergens present in the same or potentially cross-reactive materials. However, the reason for the lower frequency of LTP-driven allergies in Central/Northern Europe still remains unclear, considering no obvious substantial differences in the genetic background and the nutritional behavior (processing and time point of introduction of LTP-containing food) in the population. It is tempting to speculate that the immune system of atopic individuals in Central/Northern Europe is employed with the response to a highly immunogenic pollen, which does not express substantial amounts of LTP (e.g., birch pollen), hampering a substantial immune response to LTPs. So far, geographical differences in the role of LTPs have not been attributed to potential differences in the B and T cell epitopes in both populations.

The clinical manifestation of LTP allergy outside the Mediterranean can be grouped in diverse phenotypes, which remain challenging to predict by presence of specific IgE. There is some evidence that co-sensitization to LTP-unrelated allergens, likewise in the birch-food syndrome, transforms into less severe LTP-mediated clinical reactivity, a phenomenon sometimes described as a “protective” effect by IgE directed against allergens other than LTPs.

The prevailing opinion is that Pru p 3 displays the strongest allergenicity among the LTP family and induces sensitization to other food LTPs. The role of LTPs in the Mediterranean area can be classified as follows: (1) Pru p 3 acts as a primary sensitizer in areas with low exposure of LTP-containing pollen but shows relevant IgE cross-reactivity with LTPs from pollen and food (very frequent), (2) both Pru p 3 and pollen-LTP can act as primary sensitizers in areas with high exposure of LTP-containing pollen (olive and plane tree and mugwort, ragweed, pellitory) and possess limited IgE cross-reactivity leading to a mixed phenotype (frequent), and (3) pollen LTP act as a primary sensitizer in areas with extremely high exposure to LTP-containing pollen and no IgE cross-reactivity with Pru p 3, e.g., in olive pollen LTP mono-sensitized patients (less frequent).

The role of LTPs in the outside the Mediterranean basin is suggested as follows: (1) Pru p 3 or other food LTPs can act as a primary sensitizer in areas with low exposure of LTP-containing pollen but with high abundance of non-LTP-containing allergic pollen (preferentially birch in Central/Northern Europe) and display a strong cross-reactivity with pollen and food LTPs and (2) pollen LTPs which are genuine allergens in areas with extremely high exposure to LTP-containing pollen (e.g., mugwort pollen in Northern China) leading to secondary LTP-mediated food allergies.
